# Human and Mouse Eosinophils Differ in Their Ability to Biosynthesize Eicosanoids, Docosanoids, the Endocannabinoid 2-Arachidonoyl-glycerol and Its Congeners

**DOI:** 10.3390/cells11010141

**Published:** 2022-01-02

**Authors:** Anne-Sophie Archambault, Julyanne Brassard, Émilie Bernatchez, Cyril Martin, Vincenzo Di Marzo, Michel Laviolette, Louis-Philippe Boulet, Marie-Renée Blanchet, Nicolas Flamand

**Affiliations:** 1 Centre de Recherche de l’Institut Universitaire de Cardiologie et de Pneumologie de Québec, Département de Médecine, Faculté de Médecine, Université Laval, Quebec City, QC G1V 4G5, Canada; Anne-Sophie.archambault@criucpq.ulaval.ca (A.-S.A.); Julyanne.Brassard@ubc.ca (J.B.); Emilie.Bernatchez@criucpq.ulaval.ca (É.B.); Cyril.Martin@criucpq.ulaval.ca (C.M.); Vincenzo.dimarzo@criucpq.ulaval.ca (V.D.); Michel.Laviolette.1@ulaval.ca (M.L.); lpboulet@med.ulaval.ca (L.-P.B.); 2 Canada Excellence Research Chair on the Microbiome-Endocannabinoidome Axis in Metabolic Health (CERC-MEND), Université Laval, Quebec City, QC G1V 0A6, Canada; 3 The Biomedical Research Centre, University of British Columbia, Vancouver, BC V6T 1Z3, Canada; 4 Institut sur la Nutrition et les Aliments Fonctionnels, Centre NUTRISS, École de Nutrition, Faculté des Sciences de L’agriculture et de L’alimentation, Université Laval, Quebec City, QC G1V 0A6, Canada; 5 Joint International Unit between the Consiglio Nazionale delle Ricerche (CNR), 80078 Pozzuoli, Italy; 6 Chemical and Biomolecular Research on the Microbiome and Its Impact on Metabolic Health and Nutrition (UMI-MicroMeNu), Université Laval, Quebec City, QC G1V 0A6, Canada

**Keywords:** eosinophil, eicosanoid, docosanoid, leukotriene, prostaglandins, specialized pro-resolving mediators, endocannabinoids, asthma, arachidonic acid, docosahexaenoic acid

## Abstract

High eosinophil (EOS) counts are a key feature of eosinophilic asthma. EOS notably affect asthmatic response by generating several lipid mediators. Mice have been utilized in hopes of defining new pharmacological targets to treat asthma. However, many pinpointed targets in mice did not translate into clinics, underscoring that key differences exist between the two species. In this study, we compared the ability of human (h) and mouse (m) EOS to biosynthesize key bioactive lipids derived from arachidonic acid (AA), eicosapentaenoic acid (EPA) and docosahexaenoic acid (DHA). hEOS were isolated from the blood of healthy subjects and mild asthmatics, while mEOSs were differentiated from the bone marrow. EOSs were treated with fatty acids and lipid mediator biosynthesis assessed by LC-MS/MS. We found that hEOS biosynthesized leukotriene (LT) C_4_ and LTB_4_ in a 5:1 ratio while mEOS almost exclusively biosynthesized LTB_4_. The biosynthesis of the 15-lipoxygenase (LO) metabolites 15-HETE and 12-HETE also differed, with a 15-HETE:12-HETE ratio of 6.3 for hEOS and 0.727 for mEOS. EOS biosynthesized some specialized pro-resolving mediators, and the levels from mEOS were 9-times higher than those of hEOS. In contrast, hEOS produced important amounts of the endocannabinoid 2-arachidonoyl-glycerol (2-AG) and its congeners from EPA and DHA, a biosynthetic pathway that was up to ~100-fold less prominent in mEOS. Our data show that hEOS and mEOS biosynthesize the same lipid mediators but in different amounts. Compared to asthmatics, mouse models likely have an amplified involvement of LTB_4_ and specialized pro-resolving mediators and a diminished impact of the endocannabinoid 2-arachidonoyl-glycerol and its congeners.

## 1. Introduction

Asthma is a chronic respiratory disease characterized by airway remodeling, hyperresponsiveness and inflammation [1,2]. Eosinophils (EOS) participate in the inflammatory cascade and cause damage to the airways, further increasing inflammation by producing many pro-inflammatory effectors, notably bioactive lipids [3]. Investigating the functional roles of eosinophils is challenging due to the small number of blood eosinophils in humans. Thus, the development of mouse models was helpful to understand the pathogenesis of eosinophilic asthma and to study the role of eosinophils. These models also helped to pinpoint pharmacological targets to treat asthma, such as IL-5 or cysteinyl-leukotrienes (cysLTs) [4]. However, many pinpointed targets did not translate into clinical benefits, underscoring that key differences exist between mice and humans. For example, the inhibition of leukotriene (LT) B_4_ biosynthesis/effects in mice blocked the development/severity of the asthmatic response [5,6]. However, LTB_4_ blockade in humans had no beneficial effect on asthmatic response [7,8]. Moreover, key differences in the migration of EOS between humans (h) and mice (m) have been documented. Indeed, CCL5, CCL26 and 5-oxo-6*E*,8*Z*,11*Z*,14*Z*-eicosatetraenoic acid (5-KETE), the three most potent mediators inducing hEOS migration, are either not expressed/biosynthesized in mice or do not elicit the migration of mEOS, underscoring key differences in cellular and molecular mechanisms regulating hEOS and mEOS migration/activation [9].

Numerous bioactive lipids participate in asthmatic response and, thus, affect many aspects of the pathology [10]. Some increase inflammation by recruiting cells to the lungs, such as 5-KETE and LTB_4_, while others induce bronchoconstriction, such as cysLTs [7]. Furthermore, bioactive lipids can dampen inflammatory responses, as it is often the case of endocannabinoids [11,12] and specialized pro-resolving mediators (SPMs) [13]. Thus, it becomes a fundamental importance to better understand the similarities and differences in the ability of inflammatory cells, notably eosinophils, to biosynthesize and respond to bioactive lipids in the context of asthma.

In this paper, we compared the ability of hEOS and mEOS to biosynthesize key bioactive lipids arising from the metabolism of arachidonic acid (AA), eicosapentaenoic acid (EPA) and docosahexaenoic acid (DHA). While the obtained lipidomic profiles were not different, mediator-wise, we found striking species-dependent differences in the abilities to biosynthesize some metabolites (e.g., CysLTs, SPMs and endocannabinoid 2-arachidonoyl-glycerol (2-AG) and its EPA-derived and DHA-derived congeners). Overall, hEOSs have a greater ability to generate CysLTs and 2-AG, while mEOS generates substantially more SPMs, which likely results in different outcomes in vivo.

## 2. Materials and Methods

### 2.1. Materials

All lipids were obtained from Cayman Chemical (Ann Arbor, MI, USA). DMSO was purchased from Sigma-Aldrich (St-Louis, MO, USA). HBSS, PBS, FBS and RPMI 1640 were purchased from Wisent Laboratories (St-Bruno, QC, Canada). Lymphocyte separation medium was purchased from Corning (Corning, NY, USA). Dextran, LC-MS-grade methanol, HPLC-grade acetic acid, LC-MS-grade acetonitrile and LC-MS-grade H_2_O were purchased from Fisher Scientific (Ottawa, ON, Canada). Stem cell factor, FMS-like tyrosine kinase 3 ligand and IL-5 were purchased from Peprotech (Rocky Hill, CT, USA). WT mice were purchased from Jackson Laboratories (Bar Harbor, ME, USA).

### 2.2. Ethics

This study was approved by the local Ethic Committee (Comité d’éthique de la recherche de l’Institut universitaire de cardiologie et de pneumologie de Québec—Université Laval), and all subjects signed a consent form. Experiments involving mice were approved by the animal local ethics committee (Comité de Protection des Animaux—Université Laval), and the Canadian animal care guidelines were followed.

### 2.3. Isolation of Human Eosinophils

Human EOSs were isolated from the peripheral blood of mild asthmatics (FEV_1_ ≥ 85% of predicted value and a β_2_ agonist on demand) or healthy volunteers, as previously described [14]. In brief, 160 mL venous blood was collected in tubes containing K_3_-EDTA as anticoagulants. The platelet rich plasma and erythrocytes were discarded by centrifugation and dextran sedimentation, respectively. Granulocytes were then obtained by eliminating mononuclear leukocytes and erythrocytes using discontinuous gradient centrifugation and hypotonic lysis with sterile water, respectively. EOSs were purified by negative selection from the granulocyte preparation using anti-CD16-conjugated magnetic beads and appropriate columns (Miltenyi Biotec, Auburn, CA, USA) according to the manufacturer’s instructions. Purity and viability of eosinophils were always >95% and were assessed by Diff-Quick coloration and trypan blue exclusion, respectively. hEOSs were utilized immediately after isolation without any treatment with cytokines.

### 2.4. Isolation/Differentiation of Mouse Eosinophils

WT C57BL/6 mice were purchased from Jackson Laboratories (Bar Harbor, ME, USA) and bred in our pathogen-free animal unit. Experiments were conducted on mice between 6 and 12 weeks of age. EOSs were differentiated from bone marrow, as previously described [15], with slight modifications. Bone marrow was obtained by flushing the tibias and femurs of male and female WT mice by using a 27-gauge needle and PBS. Red blood cells were lysed with ammonium chloride, and debris were removed by using 70 μm cell strainers. Bone marrow cells (50 × 10^6^) were differentiated for 4 days in 10 mL of RPMI 1640 supplemented with 20% FBS, 100 ng/mL Stem cell factor (SCF) and 100 ng/mL FMS-like tyrosine kinase 3 ligand (FLT3L) in a 75-square centimeter flasks. At day 4, non-adherent cells were centrifuged and washed, then cultured for 4 days in RPMI supplemented with 20% FBS and IL-5 (10 ng/mL) in a new flask. From day 8 to day 14, half of the IL-5-containing media was changed every day. At day 15, bone marrow-derived mEOSs were characterized by flow cytometry and microscopy using DiffQuik coloration (HemaStain Set, Fisher Scientific, Hampton, NH, USA).

### 2.5. Isolation of Mouse Eosinophils from Bronchoalveolar Lavage (BAL)

For the isolation of lung EOS, male and female WT mice were exposed by intranasal (in) instillation to 50 μL of 1 mg/mL of house dust mite (HDM) *Dermatophagoidus pteronyssinus* freeze dried extract (Citeq Biologics, Groningen, The Netherlands) for 10 consecutive days and then euthanized at day 11. Upon euthanasia, mice were tracheotomized with an 18-gauge catheter, and a BAL was conducted by performing three separate injections/aspirations of 1 mL saline solution. In order to eliminate most macrophages, BAL cells were incubated for 2 h in RPMI +10% FBS in a flask, and non-adherent cells were collected. EOSs were enriched from non-adherent cells by negative selection using EasySep Mouse APC Positive Selection Kit II (Stemcell Technologies, Vancouver, BC, Canada) with Ly-6G-APC (BioLegend, San Diego, CA, USA), CD90.2-APC (BioLegend, San Diego, CA, USA), CD19-APC (BioLegend) and F4/80-APC (BioLegend) antibodies to remove neutrophils, T cells, B cells and macrophages, respectively. The purity of the obtained mEOS suspensions was assessed by flow cytometry and microscopy using DiffQuik coloration (HemaStain Set, Hampton, NH, USA).

### 2.6. Flow Cytometry

Bone marrow-derived mEOSs were stained with anti-mouse CD45-APC-Cy7 (BioLegend); CD11b-Pe-Cy7 (BD Biosciences, San Jose, CA, USA); CCR3-Pe-Cy7 (BioLegend); CD44-Pe-Cy7 (BioLegend); CD62-L-Pe-Cy7 (BD Biosciences); Siglec-F-APC-Cy7 (BD Biosciences); CD11c-Pe-Cy7 (BioLegend); and MHCII-Pe-Cy7 (BioLegend). Airway mEOSs were stained with CCR3-Pe-Cy7, CD45-APC-Cy7 and NK1.1-biotin (Ablab, Vancouver, Canada); CD90.2-biotin (BioLegend); CD19-biotin (BioLegend); Siglec-F-BV711 (BD Biosciences); CD11c-Pacific Blue (BioLegend); and Ly-6G-PE (BioLegend). Cells were analyzed by using a BD LSRFortessa cytometer (BD Biosciences) and FlowJo software V10 (BD, Franklin Lakes, NJ, USA).

### 2.7. Cell Stimulations

EOS suspensions (5 × 10^6^ cells/mL in HBSS containing 1.6 mM CaCl_2_) were warmed at 37 °C for 10 min. Cells were treated with AA, EPA or DHA for 15 min. Incubations were stopped by adding one volume of cold (−20 °C) MeOH containing 0.01% acetic acid and internal standards (Table 1). Samples were kept at −30 °C until the extraction of lipid mediators.

### 2.8. Extraction and Quantification of Lipid Mediators by LC-MS/MS

Lipids were extracted with solid phase extraction (SPE) cartridges (Strata-X Polymeric Reversed Phase, 60 mg/3 mL, Phenomenex, Torrance, CA, USA), as we previously described [16]. In brief, the denatured samples were centrifuged to eliminate cell debris and diluted with water to obtain a final MeOH concentration of 10% and acidified (pH 3) with acetic acid. Samples were then loaded onto the SPE cartridges, eluted with 1 mL of MeOH, dried under a stream of nitrogen and reconstituted with 25 μL of solvent A (H_2_O containing 1 mM ammonium hydroxide and 0.05% of acetic acid) and 25 μL of solvent B (MeCN/H_2_O, 95/5, *v/v* containing 1 mM of ammonium hydroxide and 0.05% of acetic acid). Samples (40 μL) were co-injected with 40 μL H_2_O onto an HPLC column (Kinetex C8, 150 × 2.1 mm, 2.6 μm, Phenomenex) and eluted at a flow rate of 0.4 mL/min with a discontinuous gradient from 35% to 75% of solvent B in 10 min, from 75% to 95% in 10 s and held to 95% for 5 min. The HPLC system was interfaced with the electrospray source of a Shimadzu 8050 triple quadrupole mass spectrometer, and mass spectrometric analysis was performed in the negative or positive ion modes by using multiple reaction monitoring for the specific mass transition of the metabolites, as shown in Table 1. Importantly, our analytical method does not discriminate between R and S isomers of some compounds, notably the HETEs, HDHAs and possibly some dihydroxylated products such as PDX (vs. PD1) and Mar1 (vs. 7(S),14(S)-DiHDHA).

### 2.9. Statistical Analysis

Statistical analyses were performed by using GraphPad Prism 8 software. Data were log-transformed, and individual *t*-tests for each mediator were performed. The normality assumption was verified with the Shapiro–Wilk test. *p*-values < 0.05 were considered significant. * *p* < 0.05; ** *p* < 0.01; *** *p* < 0.001; **** *p* < 0.0001.

## 3. Results

### 3.1. Characterization of Mouse Bone Marrow-Derived Eosinophils

The first step to ensure an adequate comparison between hEOS and mEOS was to secure the access of mEOS given that it was not possible to isolate them from blood due to their low number. As such, we obtained mEOS by differentiating the bone marrow hematopoietic precursors of mice. We opted for the established differentiation protocol described in Material and Methods that allows the generation of a reasonable number of mEOS [17]. These EOSs are fully differentiated after 14 days with a donut-shaped nucleus and the expression of both CCR3 and Siglec-F [17,18,19]. Furthermore, bone-marrow-derived mEOSs differentiated using this protocol are functional, possessing the ability to migrate, to produce cytokines and to participate in allergic asthmatic responses in mice [15,20,21]. In our hands, more than 95% of differentiated cells were EOS based on cell morphology (Figure S1A), as well as CCR3 and Siglec-F expression. Indeed, the analysis of differentiated cells by flow cytometry confirmed the expression of CD45, CD11b, CD44, Siglec-F and CCR3 (Figure S1B,C), which are markers expressed by mEOS [22,23,24]. CD11c and MHCII molecules were almost absent on mEOS, ruling out unspecific differentiation into macrophages or dendritic cells [25,26].

### 3.2. Biosynthesis of Eicosanoids by hEOS and mEOS

**Figure 1:**
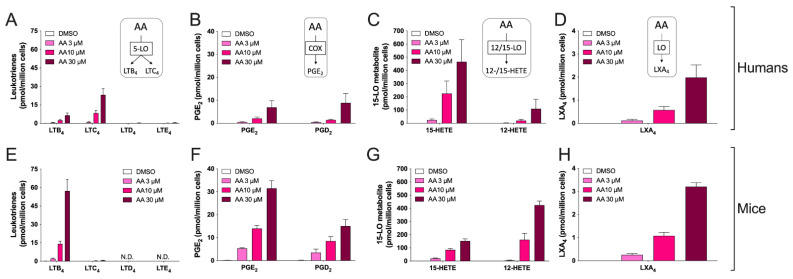
Biosynthesis of AA-derived eicosanoids from human and mouse eosinophils. EOS suspensions (5 × 10^6^ cells/mL, 37 °C) were treated with DMSO or increasing concentrations of AA for 15 min. Incubations were stopped by adding one volume of cold (−20 °C) MeOH containing internal standards. Samples were then processed and analyzed for eicosanoids by LC-MS/MS, as described in Materials and Methods. (**A**–**D**) Results are the mean (±SEM) of 6 independent experiments using human eosinophils from asthmatic donors. (**E**–**H**) Results are the mean (±SEM) of 7 independent experiments using bone marrow-derived mouse eosinophils.

In the next series of experiments, we compared the ability of hEOS and mEOS to biosynthesize leukotrienes in response to exogenously added AA. We chose exogenously added AA because it allows bypassing phospholipases A_2_, which are responsible to release fatty acids. This provided a model in which we can assess the biosynthesis of eicosanoids in both species with the same substrate concentrations. We tested three AA concentrations (3, 10 and 30 µM) to ensure that our experimental approach would result in the detection of all major metabolites biosynthesized by these cells. When treated with 10 µM AA, the sum of LTs was not different between species (*p* = 0.32, *t*-test). Under that setting, hEOS biosynthesized both LTC_4_ and LTB_4_ with a LTC_4_/LTB_4_ ratio of 5.151 ± 1.556 (Figure 1A) while bone marrow-derived mEOS mainly biosynthesized LTB_4_ with only minimal quantities of LTC_4_, resulting in an LTC_4_/LTB_4_ ratio of 0.027 ± 0.009 (Figure 1E). The inability of mEOS to biosynthesize large amounts of LTC_4_ was not the consequence of increased metabolism of LTC_4_ into LTD_4_ and/or LTE_4_ as the latter two were not detected in AA-treated mEOS despite the high sensitivity of our method (Figure 1E and Table 1**).** Of note, both hEOS and mEOS produced low levels of LXA_4_, and no difference was observed between the two species (Figure 1D,H).

Prostaglandins (PG) have documented roles in asthma. Indeed, PGE_2_ inhibits leukocyte functions and enhances bronchodilation [27,28,29], while PGD_2_ mainly recruits and activates key leukocytes involved in asthmatic responses, notably EOS [9,14,30]. In addition, EOSs were documented as a source of these cyclooxygenase-derived metabolites, notably PGE_2_ [31,32]. We thus quantitated the levels of PGE_2_, PGD_2_ and the PGI_2_ metabolite 6-keto-PGF_1α_ and found that hEOS and mEOS biosynthesized PGE_2_ and PGD_2_ but not 6-keto-PGF_1α_ (detection limit of 50 fmol). While both hEOS and mEOS biosynthesized comparable levels of PGD_2_, hEOS biosynthesized significantly less PGE_2_ than mEOS (*p* < 0.0001 for AA 10 μM, multiple *t*-tests, Figure 1B,F). Unfortunately, our analytical method did not include the analysis of the thromboxane (TX) synthase metabolites TXA_2_ (assessed by quantitating TXB_2_) and 12-HHTrE at the time of analysis, thereby representing a limitation of this study.

Over the last decades, it has become clear that the 15-lipoxygenase (15-LO) pathway participates in the asthmatic response. Accordingly, 15-LO-1 expression is increased in asthma [33], and its metabolites increased in the sputum of asthmatic patients [33,34]. 15-LO-1 can metabolize fatty acids, endocannabinoids and phospholipids [35]. The murine ortholog of 15-LO-1 is 12/15-LO [36], and its absence protects it from experimental asthma [37,38]. Differences in enzyme activity between recombinant 15-LO-1 and 12/15-LO have previously been reported [36,39]. Consequently, we compared the ability of hEOS and mEOS to biosynthesize 15-HETE and 12-HETE. In response to exogenously added AA, hEOS mainly biosynthesized 15-HETE (Figure 1C), while mEOS mainly biosynthesized 12-HETE (Figure 1G). 15-HETE/12-HETE ratios obtained with EOS stimulated with 10 μM AA were 6.319 ± 0.465 for hEOS and 0.727 ± 0.154 for mEOS (mean ± SEM, *t*-test, *p* = 0.009). These differences were expected and are in line with the previously documented differences in AA utilization by 15-LO-1 and 12/15-LO in cellulo and in vitro [36,39,40].

**Figure 2:**
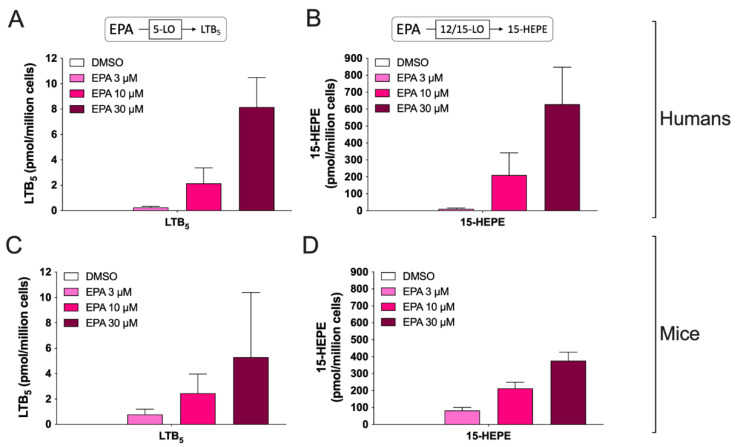
Biosynthesis of EPA-derived eicosanoids from human and mouse eosinophils. EOS suspensions (5 × 10^6^ cells/mL, 37 °C) were incubated with DMSO or increasing concentrations of EPA for 15 min. Incubations were stopped by adding one volume of cold (−20 °C) MeOH containing the internal standards. Samples were then processed and analyzed for eicosanoids by LC-MS/MS as described in Materials and Methods. (**A**,**B**) Results are the mean (±SEM) of 6 independent experiments using human eosinophils from asthmatic donors. (**C**,**D**) Results are the mean (±SEM) of 7 independent experiments using bone marrow-derived mouse eosinophils.

In another series of experiments, we investigated the biosynthesis of eicosanoids derived from the ω-3 fatty acid eicosapentaenoic acid (EPA). In that regard, we only investigated the biosynthesis of LTB_5_, resolvin (Rv) E_1_ and 15-hydroxy-eicosapentaenoic acid (15-HEPE). Both hEOS and mEOS produced LTB_5_ and 15-HEPE after treatment with EPA (Figure 2). Indeed, while we observed higher levels of LTB_5_ in hEOS vs. mEOS at concentrations of EPA of 10 μM and 30 μM, this difference only reached statistical significance at a concentration of 30 μM (*p* = 0.03, Mann–Whitney test). 15-HEPE levels were not significantly different between hEOS and mEOS. Of note, RvE_1_ was not detected in hEOS nor mEOS (detection limit of 50 fmol) even after a treatment with 30 µM EPA, indicating that EOSs are not a good source of that metabolite Unfortunately, we did not analyze the biosynthesis of 18(R)-HEPE in these experiments, the latter being considered the RvE1 precursor [13]. Furthermore, we did not analyze the levels of RvE3 even though mEOSs are capable of biosynthesizing it [41] due to the lack of a commercially available standard.

### 3.3. Biosynthesis of Docosanoids by hEOS and mEOS

**Figure 3:**
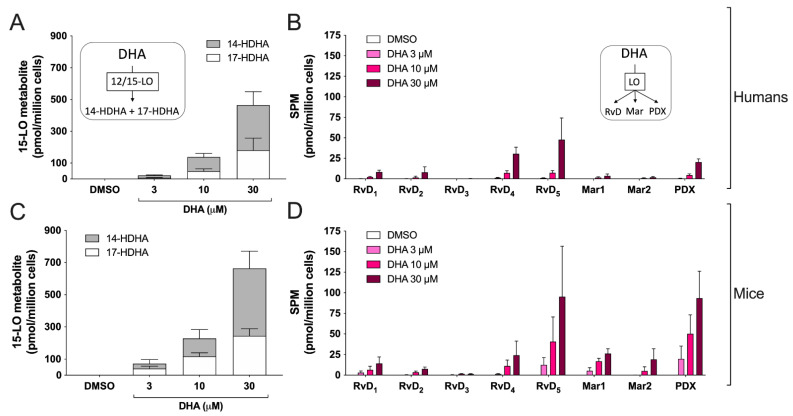
Biosynthesis of docosanoids by human and mouse eosinophils. EOS suspensions (5 × 10^6^ cells/mL, 37 °C) were incubated with DMSO or increasing concentrations of DHA for 15 min. Incubations were stopped by adding one volume of cold (−20 °C) MeOH containing the internal standards. Samples were then processed and analyzed for docosanoids by LC-MS/MS as described in Materials and Methods. (**A**,**B**) Results are the mean (± SEM) of 5 independent experiments using human eosinophils from asthmatic donors. (**C**,**D**) Results are the mean (±SEM) of 7 independent experiments using bone marrow-derived mouse eosinophils.

We next evaluated the metabolism of DHA by EOS. When EOSs were treated with 30 µM DHA, hEOS and mEOS biosynthesized 14-HDHA and 17-HDHA with respective 14-HDHA:17-HDHA ratios of 1.57 ± 0.81 and 1.73 ± 0.54 (Figure 3A,C). However, lower concentrations of DHA resulted in significant differences in the 14-HDHA:17-HDHA ratio. Indeed, 14-HDHA:17-HDHA ratios were 2.15 ± 0.74 for hEOS and 0.75 ± 0.67 for mEOS when cells were treated with 3 µM DHA, while they were 1.82 ± 0.72 for hEOS and 0.94 ± 0.50 for mEOS at 10 µM DHA. In addition to 14-HDHA and 17-HDHA, DHA can also be metabolized into a wide variety of SPMs, including D-series resolvins (RvD), maresins (Mar) and protectin D_1_ (PDX) [13]. To that end, we went on to confirm that hEOS and mEOS could biosynthesize all these SPMs (Figure 3B,D). When treating EOS with 10 μM DHA, the levels of most individual SPM tended to be lower in hEOS vs. mEOS, and this difference reached statistical significance for Mar1 (*p* = 0.0002, *t*-test) and PDX (*p* = 0.006, *t*-test). However, we observed that when combined, mEOS produced more SPMs than hEOS (nine times more at 10 µM DHA), supporting the concept that mEOSs are more prone to biosynthesize SPMs than compared to hEOS.

### 3.4. Biosynthesis of the Endocannabinoid 2-AG and Its Congeners by hEOS and mEOS

**Figure 4:**
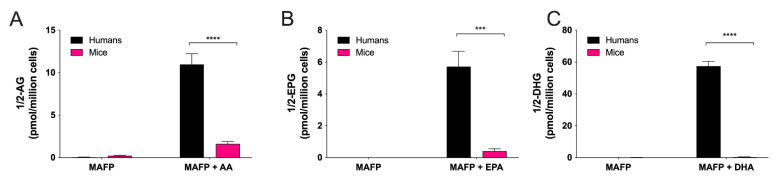
Biosynthesis of the endocannabinoid 2-AG and its congeners by human and mouse eosinophils. EOS suspensions (5 × 10^6^ cells/mL, 37 °C) were treated 1 μM of MAFP for 5 min then incubated with DMSO or 10 μM of (**A**) AA, (**B**) EPA, or (**C**) DHA for 15 min. Incubations were stopped by adding one volume of cold (−20 °C) MeOH containing the internal standards. Samples were then processed and analyzed for 2-AG and related mediators by LC-MS/MS as described in Materials and Methods. Results are the mean (±SEM) of 4 independent experiments using human eosinophils from asthmatic donors and 5 independent experiments using bone marrow-derived mouse eosinophils. *p*-values were obtained as described in Materials and Methods. *** *p* < 0.001, **** *p* < 0.0001.

Endocannabinoids are eicosanoids exerting numerous anti-inflammatory effects, at least in mice [11,12]. The two endocannabinoids, 2-arachidonoyl-glycerol (2-AG) and *N*-arachidonoyl-ethanloamine (AEA), contain an AA moiety in their structure and are part of two major lipid classes belonging to endocannabinoidome, monoacylglycerols and the *N*-acyl-ethanolamines [42]. Importantly, 2-AG is rapidly hydrolyzed into AA by hEOS [14,43]. However, we can prevent such hydrolysis with serine hydrolase inhibitors such as MAFP, which allows correctly assessing unsaturated fatty acid-induced biosynthesis of 2-AG and other monoacylglycerols [44]. As expected, in the presence of MAFP, hEOS biosynthesized 2-AG, 2-EPG and 2-DHG in response to AA, EPA and DHA, respectively (Figure 4), to comparable levels of what we previously documented [44]. mEOS also biosynthesized these three monoacylglycerols in response to exogenously added fatty acid but to a much lower extent (Figure 4), indicating that mEOSs display a significantly lower monoacylglycerol biosynthetic capability than hEOS. Compared to mEOS, hEOS produced 7-times more 2-AG, 14-times more 2-EPG and 98-times more 2-DHG.

### 3.5. Impact of the Activation of Cells by PAF in hEOS and mEOS

**Figure 5:**
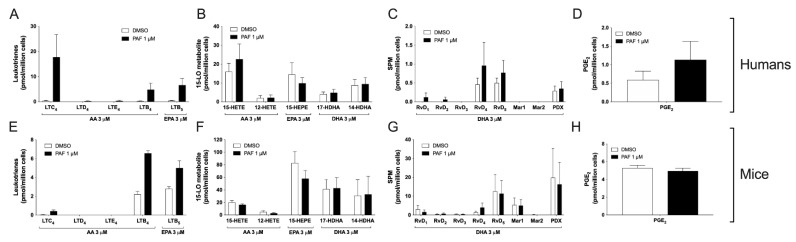
Impact of PAF on eicosanoid and docosanoid biosynthesis by human and mouse eosinophils. EOS suspensions (5 × 10^6^ cells/mL, 37 °C) were stimulated 15 min with 1 μM PAF and 3 μM of AA, EPA or DHA. Incubations were stopped by adding 1 volume of cold (−20 °C) of MeOH containing the internal standards. Lipids were extracted and quantified by LC-MS/MS as described in Materials and Methods. (**A**–**D**) Results are the mean (±SEM) of 5 independent experiments using human eosinophils from asthmatic donors. (**E**–**H**) Results are the mean (±SEM) of 7 independent experiments using bone marrow-derived mouse eosinophils.

PAF is a known activator of lipid mediator synthesis. In neutrophils, PAF increases AA-induced LT biosynthesis, most likely by increasing intracellular calcium concentration, thereby activating 5-LO [14,45]. Given the low levels of LTC_4_ we detected following the treatment of mEOS with AA (Figure 1), we tested whether the activation of 5-LO with PAF could stimulate the production of this mediator. We first treated mEOS and hEOS with 1 µM PAF. Under that setting, PAF did not induce the biosynthesis of eicosanoids (data not shown). However, when we treated EOS suspensions with the combination of PAF and AA (1 and 3 μM, respectively), we observed that PAF increased the AA-induced biosynthesis of LTC_4_ and LTB_4_ and the EPA-induced synthesis of LTB_5_ by hEOS (Figure 5A). PAF also increased the biosynthesis of LTC_4_, LTB_4_ and LTB_5_ by mEOS (Figure 5E). However, the levels of cysLT remained lower in mEOS compared to hEOS, confirming that they do not biosynthesize these metabolites to the same extent as hEOS. PAF had no impact on the biosynthesis of 15-LO metabolites (Figure 5B,F), SPMs (Figure 5C,G) or the COX metabolite PGE_2_ (Figure 5D,H), underscoring that 15-LO and COX pathways are not modulated by this autacoid when exogenous substrates are present.

### 3.6. Differences between Eosinophils Isolated from Mild Asthmatics and Healthy Subjects

**Figure 6:**
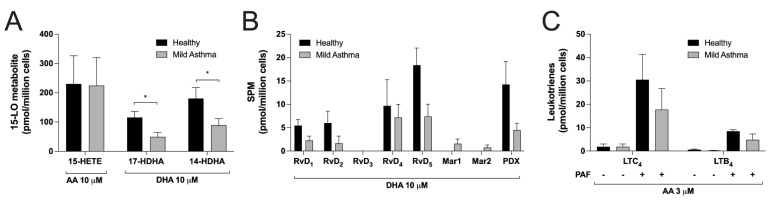
Comparison between eosinophils from healthy and asthmatic donors. EOS suspensions (5 × 10^6^ cells/mL, 37 °C) were treated with (**A**) 10 µM AA or DHA, (**B**) 10 µM DHA or (**C**) the combination of 1 μM PAF and 3 μM AA for 15 min. Incubations were stopped by adding one volume of cold (−20 °C) MeOH containing the internal standards. Samples were processed and analyzed for eicosanoids and docosanoids content by LC-MS/MS, as described in Materials and Methods. Results are the mean (±SEM) of 5 independent experiments using human eosinophils from asthmatic donors and 5 independent experiments using human eosinophils from healthy donors. Data from asthmatic donors are the same as those presented in Figure 1, Figure 3 and Figure 5. *p*-values were obtained as described in Materials and Methods. * *p* < 0.05.

Data from Figure 1, Figure 2, Figure 3, Figure 4 and Figure 5 were obtained using hEOS from mild asthmatics to ensure we had a sufficient number of EOS to perform experiments with each fatty acid. However, in order to validate our findings, we compared the ability of hEOS from mild asthmatics with those from healthy volunteers in a limited number of experimental conditions. While we observed a trend toward lower levels of eicosanoids, docosanoids and SPMs in mild asthmatic vs. healthy donors, this difference only reached statistical significance for 14-HDHA and 17-HDHA (Figure 6A,B). The combination of PAF/AA had the same impact on LT biosynthesis regardless of disease status (Figure 6C).

### 3.7. Differences between Bone Marrow-Derived and Lung Eosinophils from Mice

**Figure 7:**
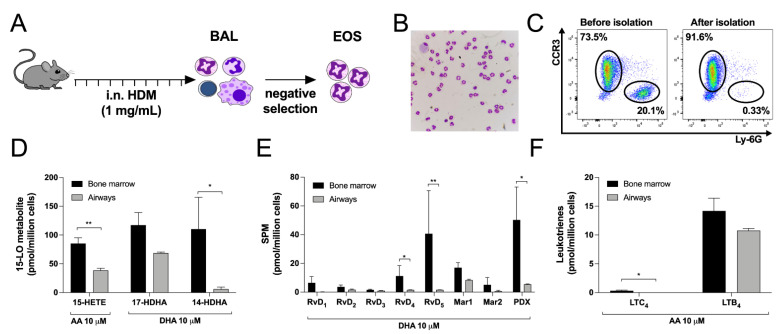
Comparison between eosinophils isolated from the airways of HDM-treated mice and eosinophils derived from the bone marrow. (**A**) Experimental design to isolate mouse eosinophils from the airways of HDM-treated mice. (**B**) Morphology of airway cells after eosinophils enrichment determined by DiffQuik staining. (**C**) Purity of airway EOS before and after negative selection. (**D**–**F**) Eosinophils (5 × 10^6^ cells/mL, 37 °C) were treated with (**D**) 10 µM AA or 10 µM DHA and (**E**) 10 µM DHA or (**F**) 10 µM AA for 15 min. Incubations were stopped by adding one volume of cold (−20 °C) MeOH containing the internal standards. Samples were processed and analyzed for eicosanoids and docosanoids content by LC-MS/MS, as described in Materials and Methods. Results are the mean (±SEM) of 7 independent experiments using bone marrow-derived mouse eosinophils and 4 independent experiments using mouse eosinophils isolated from the airways. Data from bone marrow derived eosinophils are the same as those presented in Figure 1, Figure 3 and Figure 5. *p*-values were obtained as described in Materials and Methods. * *p* < 0.05, ** *p* < 0.01.

Due to the face that data from Figure 1, Figure 2, Figure 3, Figure 4 and Figure 5 were generated using bone marrow-derived mEOS, we next assessed if we would obtain the same biosynthetic profile using lung mEOS. Thus, we utilized a model of HDM-exposed mice that develops severe eosinophilia in the lungs [46] (Figure 7A). After enrichment, mEOS isolated from the airways of HDM-exposed mice represented 86.74 ± 1.61 of our suspension, the major contaminants being macrophages (4.68 ± 0.15%), lymphocytes (including B, T and NK cells) (7.40 ± 1.14%) and neutrophils (1.21 ± 0.44%) (Figure 7B,C). When compared to bone marrow-derived mEOS, the ability of airway mEOS to biosynthesize eicosanoids and docosanoids was lower and reached statistical significance for 15-HETE, 14-HDHA, RvD4, RvD5, PDX and LTC_4_ (Figure 7D–F). Of note, Cys-LT biosynthesis was even lower despite the location of mEOS in the airways.

## 4. Discussion

EOS infiltration into the airways is a key feature of asthma. Many mouse models of asthma were developed over the years to identify molecular targets that could limit this infiltration. To that end, eosinophilic asthma models have been developed in BALB/c and C57BL/6 mice (the latter background being often utilized for transgenic mice), and some differences were identified between asthmatics and experimental mice. Thus, understanding what differs between these two species remains crucial to identify the right targets that could be translated into the clinic. In that regard, we herein investigated the ability of hEOS and mEOS to biosynthesize eicosanoids, docosanoids and endocannabinoids when the involvement of phopholipases A_2_ is bypassed by exogenous substrates. While EOS from both species could biosynthesize the same lipid mediators, we observed significant differences in the levels of some mediators. Compared to mEOS from C57BL/6 mice, hEOS biosynthesized (1) more LTC_4_ and less LTB_4_; (2) more 15-HETE than 12-HETE; (3) less PGE_2_; (4) less SPMs; and (5) more endocannabinoid 2-AG and its EPA and DHA-derived congeners.

The LT biosynthetic profile between hEOS and mEOS is the first major difference we observed. Indeed, the LTC_4_/LTB_4_ ratio of hEOS was 5.151 ± 1.556, while that of mEOS was 0.027 ± 0.009. The biosynthesis of LTB_4_ by hEOS was expected, as they express LTA_4_ hydrolase and can generate LTB_4_ in response to some stimuli [47]. However, LTB_4_ was the major LT biosynthesized by mEOS, with a limited amount of cysLTs even when activated with the combination of AA and PAF, which mobilizes Ca^2+^ ions (a potent stimulator of 5-LO activity) from intracellular stores. Importantly, the levels of cysLTs obtained from our mEOS preparations are comparable to those reported for isolated mEOS previously [48,49]. Of note, murine LTC_4_ synthase has a lower affinity for LTA_4_ than the human enzyme [50], which could explain, at least in part, the striking difference in LTC_4_ biosynthesis between mEOS and hEOS. Furthermore, increasing the expression of LTC_4_ synthase in mice better mimicked aspirin-exacerbated respiratory disease [51] despite the fact that LTD_4_ is not a very potent bronchoconstrictor in mice compared to humans [52]. Altogether, our data are in line with the concept that mEOS-related inflammatory diseases are more likely driven by LTB_4_ rather than by cysLTs. This would nicely explain why the blockade of LTB_4_ receptor 1 efficiently prevents asthmatic responses in mice but not in humans [5,6,8].

Another key difference in the eicosanoid profile was the contrasting levels of 12-HETE and 15-HETE. Indeed, we not only found differences in their absolute amount, but we also observed that the 15-HETE/12-HETE ratio, using 10 µM AA, was 86:14 for hEOS while that of mEOS was 42:58 (Figure 1C,F). This is in line with previously reported 15-HETE/12-HETE ratios for the two species [36,53]. The precise roles of 15-HETE and 12-HETE in asthma remain ill defined. In addition, these lipid mediators do not bind to the same receptors, and they can be further metabolized into other lipid mediators with potential biological activities [54]. Importantly, 15-LO-1 expression and 15-HETE levels increased in severe eosinophilic asthmatics [33]. Furthermore, individuals having a loss-of-function mutation in the *alox15* gene have a lower incidence of nasal polyps and chronic rhinosinusitis [55], underscoring a deleterious role of 15-LO-1 in allergic diseases, possibly including asthma. In mice, the deletion of 12/15-LO protects them from the development of an asthmatic phenotype in response to ovalbumin [37] but increases IL-33-induced inflammation, which coincides with decreased 12-HETE and 15-HETE levels [56]. Importantly, the 15-LO pathway is also involved in the biosynthesis of other lipid mediators from arachidonic acid, notably eoxins and lipoxins. While eoxins can be biosynthesized by hEOS [57], our experimental setup did not allow us to detect them. Furthermore, a very low but comparable amount of LXA_4_ was detected in both hEOS and mEOS (Figure 1D,H), underscoring that both eoxins and lipoxins were not major lipid mediators biosynthesized by EOS in the experimental model we utilized.

The 15-LO pathway also results in the biosynthesis of numerous docosanoids, notably protectins, maresins and the D-series resolvins, which are members of the ever-expanding class of specialized pro-resolving mediators (SPMs [13]). Therefore, we also assessed the metabolism of DHA, which again differed between hEOS and mEOS. First, we confirmed that hEOS and mEOS both biosynthesized 17-HDHA and 14-HDHA, which are often regarded as SPM precursors, but in different proportions. For hEOS, the 17-HDHA:14-HDHA ratio (at 10 μM of DHA) was 35:65, in line with previously reports with either human recombinant 15-LO-1 or hEOS [36,40]. As for mEOS, they produced 17-HDHA and 14-HDHA in a 52:48 ratio. This is intriguing as previous studies reported that mouse recombinant 12/15-LO solely biosynthesized 14-HDHA in response to DHA [36,39]. Furthermore, a high 17-HDHA:14-HDHA ratio was found in mEOS isolated from the airways (Figure 7). The presence of 17-HDHA is difficult to interpret and might reflect the expression of another 15-LO in mEOS, which will require additional investigation, but this is in line with a recent study detecting this lipid mediator in mice [56].

Interestingly, the profile of SPMs we obtained is clearly reminiscent of what we found in the lungs of COVID-19 patients [58], although Mar1 and Mar2 were not detected in the latter study. This points out to a possible involvement of hEOS in the biosynthesis of SPMs during COVID-19 and/or it mirrors the biosynthetic ability/preference of 15-LO-1 toward DHA. Importantly, the biosynthesis of SPMs was significantly higher in mEOS vs. hEOS (*p* = 0.02 and 0.04 for 3 and 10 µM DHA, respectively). This indicates that mice (or at least their EOS) have a greater capacity to biosynthesize docosanoids and that SPM tone and effects might be more important in this species. As for the biosynthesis of the EPA-derived SPM RvE1 (chemically an 18-OH-LTB_5_), this could not be detected here following a treatment with up to 30 µM EPA, indicating that EOSs are not a good source or RvE1 in both species.

We must point out that the model we chose to assess the ability of hEOS and mEOS to biosynthesize eicosanoids and docosanoids included their fatty acid precursors (AA and DHA respectively), alone or in combination with PAF, which mobilizes intracellular Ca^2+^ ions and, thus, increases 5-LO activity. Eicosanoids and docosanoids can also be obtained, to some extent, with the pharmacological agonist A23187 or other G-protein-coupled receptor agonists alone or in combination with cytokines such as IL-5. While this might be seen as a limitation, we believe that our model has the quality of assessing the biosynthetic ability of both species at equivalent substrate concentrations. The following steps will be to determine whether species differences exist with other agonists, which will probably be the case depending on the cells’ abilities to respond to the agonists, keeping in mind that other mechanisms (intensity of Ca^2+^ release, phospholipids AA and DHA content; phospholipase involved) might explain those differences.

We recently documented a new biosynthetic pathway by which human myeloid leukocytes, including hEOS, responded to polyunsaturated fatty acid by generating significant levels of the endocannabinoid 2-AG and its congeners [44]. To that end, we observed a striking difference in the ability of hEOS and mEOS to biosynthesize the endocannabinoid 2-AG and other monoacylglycerols (2-EPG and 2-DHG) in response to polyunsaturated fatty acids. While the levels of monoacylglycerols we obtained for hEOS are comparable to our previous study [44], those obtained in mEOS were significantly lower than in hEOS (*p*-value < 0.0001 for 2-AG, 2-EPG and 2-DHG). This suggests that mEOSs are less likely to generate the endocannabinoid 2-AG and its congeners than hEOS. This finding also indicates that a plausible and possibly very important bias exists between mice and humans regarding the ability of EOS (and possibly other leukocytes) to regulate the inflammatory cascade via the endocannabinoid system, notably via the CB_2_ receptor [12]. Caution should, thus, be used when investigating the involvement of endocannabinoids and related mediators as anti-inflammatory effectors in mice (e.g., in *mgll*-deficient mice, which exhibit higher levels of 2-AG and its congeners) as it might underestimate the potential of mouse leukocytes as anti-inflammatory effectors compared to human leukocytes.

We also investigated if there were any major differences in the ability of EOS to biosynthesize the lipid mediators investigated herein in asthma. While the levels of some eicosanoids and docosanoids tended to be lower in hEOS from mild asthmatics, this difference only reached statistical significance for 14-HDHA and 17-HDHA (Figure 6), indicating that the ability of hEOS to biosynthesize eicosanoids and docosanoids is not impaired in mild asthma when AA and DHA were available. In contrast, while the lipid mediator profile was the same between bone marrow-derived mEOS and airway mEOS following a house-dust mite challenge, we observed some differences between the two. In fact, mEOS from the airways biosynthesized less LTC_4_, 15-HETE, 14-HDHA and docosanoids than bone-marrow-derived mEOS. This could be explained by the fact that in airway-mEOS, the biosynthesis of these mediators is decreased by inhibitory phosphorylations affecting the 12/15-LO and LTC_4_ synthase or by a decreased expression of these enzymes. These observations are in line with other studies that reported a decrease in 15-LO metabolite levels in blood EOS from nasal polyps or severe asthmatics [59,60] and in the lungs of LPS-treated mice [61], possibly pointing to decreased EOS 15-LO activity in EOS in a context of acute disease. However, this will need further and more detailed investigations examining how this can relate to human disease, keeping in mind that we did not observe decreased 15-HETE levels in hEOS from mild asthmatics while 15-HETE levels were previously documented as increased in sputum samples of severe eosinophilic asthmatics [33]. Finally, 12/15-LO-deficient mice have a worsen inflammatory state in the airways in a model involving IL-33 [56]. While not particularly addressing this issue herein, our data nonetheless support the concept of a possible involvement of decreased SPM biosynthesis in that model.

One limitation of our study is the fact that we compared blood eosinophils from humans and bone marrow-derived mouse eosinophils. While the perfect comparison would be to use EOS from the blood of both species, it was not possible to obtain enough eosinophils from the blood of mice to conduct all experiments included in this study. We thus opted to use a previously described protocol to generate a high number of pure bone marrow-derived EOS [17]. These EOS are fully differentiated after 14 days with a typical donut-shaped nucleus (Figure S1A) [17,18] and the expression of both CCR3 and Siglec-F (Figure S1B) [17,19]. Other studies also showed that these bone-marrow-derived EOSs are fully functional, possessing the ability to migrate, to produce cytokines and to participate in allergic asthmatic responses in mice [15,20,21]. In order to further confirm the validity of our comparison, we isolated airway EOS from mice and obtained very similar patterns of lipid biosynthesis between bone marrow-derived mEOS and airway mEOS, confirming that the major differences we observe between mEOS and hEOS are not the consequences of a defect in mEOS differentiation but rather a consequence of a difference between the species.

In conclusion, we show that while both hEOS and mEOS can biosynthesize the same lipid mediators, and the observed levels differ between the two species. The key differences are the contrasting levels of LTs, SPMs and endocannabinoid 2-AG and its congeners. While the roles of LTC_4_ and LTB_4_ are well understood in asthma, the importance of SPMs and endocannabinoids remains to be explored in depth. This is important given that SPMs can downregulate the inflammatory process and even promote its resolution while 2-AG can influence the inflammatory cascade in both directions [11]. Additional studies will, thus, be needed to assess if the differences we highlighted in this study impact the roles of EOS in mouse models of asthma, thereby providing an important bias for the translation of mice knowledge into the clinic. This will be crucial for the development of future anti-inflammatory and/or pro-resolving treatments.

## Figures and Tables

**Table 1:** Specific mass transitions of the lipid mediators.

Lipid	Internal Standard Used	Q1 → Q3	Retention Time (min)	Detection Limit (fmol)
LTB_4_-d_4_	-	339.30 → 197.20	7.39	-
15-HETE-d_8_	-	327.20 → 226.20	10.20	-
1-AG-d_5_	-	384.50 → 287.20	12.64	-
RvD_2_-d_5_	-	380.40 → 175.20	5.31	-
PGE_2_-d_4_	-	355.20 → 275.35	5.04	-
LTC_4_	LTB_4_-d_4_	624.30 → 272.20	5.69	50
LTD_4_	LTB_4_-d_4_	495.30 → 176.85	5.38	50
LTE_4_	LTB_4_-d_4_	438.30 → 333.20	6.28	50
LTB_4_	LTB_4_-d_4_	335.30 → 195.25	7.47	50
LTB_5_	LTB_4_-d_4_	333.40 → 195.25	6.62	50
15-HETE	15-HETE-d_8_	319.40 → 219.30	9.76	50
12-HETE	15-HETE-d_8_	319.10 → 179.25	10.11	50
15-HEPE	15-HETE-d_8_	317.40 → 219.25	9.05	50
17-HDHA	15-HETE-d_8_	343.50 → 281.30	10.43	50
14-HDHA	15-HETE-d_8_	343.50 → 281.30	10.59	50
2-AG	1-AG-d_5_	379.30 → 287.25	12.54	25
2-DHG	1-AG-d_5_	403.20 → 311.20	12.57	50
2-EPG	1-AG-d_5_	377.10 → 285.25	11.74	50
RvD_1_	RvD_2_-d_5_	375.40 → 141.10	5.36	50
RvD_2_	RvD_2_-d_5_	375.40 → 175.20	5.36	50
RvD_3_	RvD_2_-d_5_	375.40 → 147.20	5.15	50
RvD_4_	RvD_2_-d_5_	375.40 → 101.05	6.34	50
RvD_5_	RvD_2_-d_5_	359.40 → 199.25	7.51	50
RvE1	RvD_2_-d_5_	349.30 → 195.20	3.53	50
Maresin 1	RvD_2_-d_5_	359.40 → 177.25	7.42	50
Maresin 2	RvD_2_-d_5_	359.40 → 221.05	8.20	50
PDX	RvD_2_-d_5_	359.30 → 153.15	7.48	50
6-keto-PGF_1α_	PGE_2_-d_4_	369.30 → 163.10	3.42	50
PGE_2_	PGE_2_-d_4_	351.20 → 271.15	5.08	5
PGD_2_	PGE_2_-d_4_	351.30 → 271.20	5.31	5

## Data Availability

The data presented in this study are contained within the article or Supplementary Materials.

## Supplementary Materials

The following are available online at https://www.mdpi.com/article/10.3390/cells11010141/s1, Figure S1: Flow cytometry analysis of mouse bone marrow-derived eosinophils (mEOS).
