# Supplementary material for: Human and Mouse Eosinophils Differ in Their Ability to Biosynthesize Eicosanoids, Docosanoids, the Endocannabinoid 2-Arachidonoyl-glycerol and Its Congeners
Source: Cells. 2022 Jan 2;11(1):141. doi: 10.3390/cells11010141 (PMC8750928; doi:10.3390/cells11010141)
Supplement: Supplementary file 1 [file cells-11-00141-s001.zip › cells-1495199-supplementary.pdf]

**Supplementary Figure**

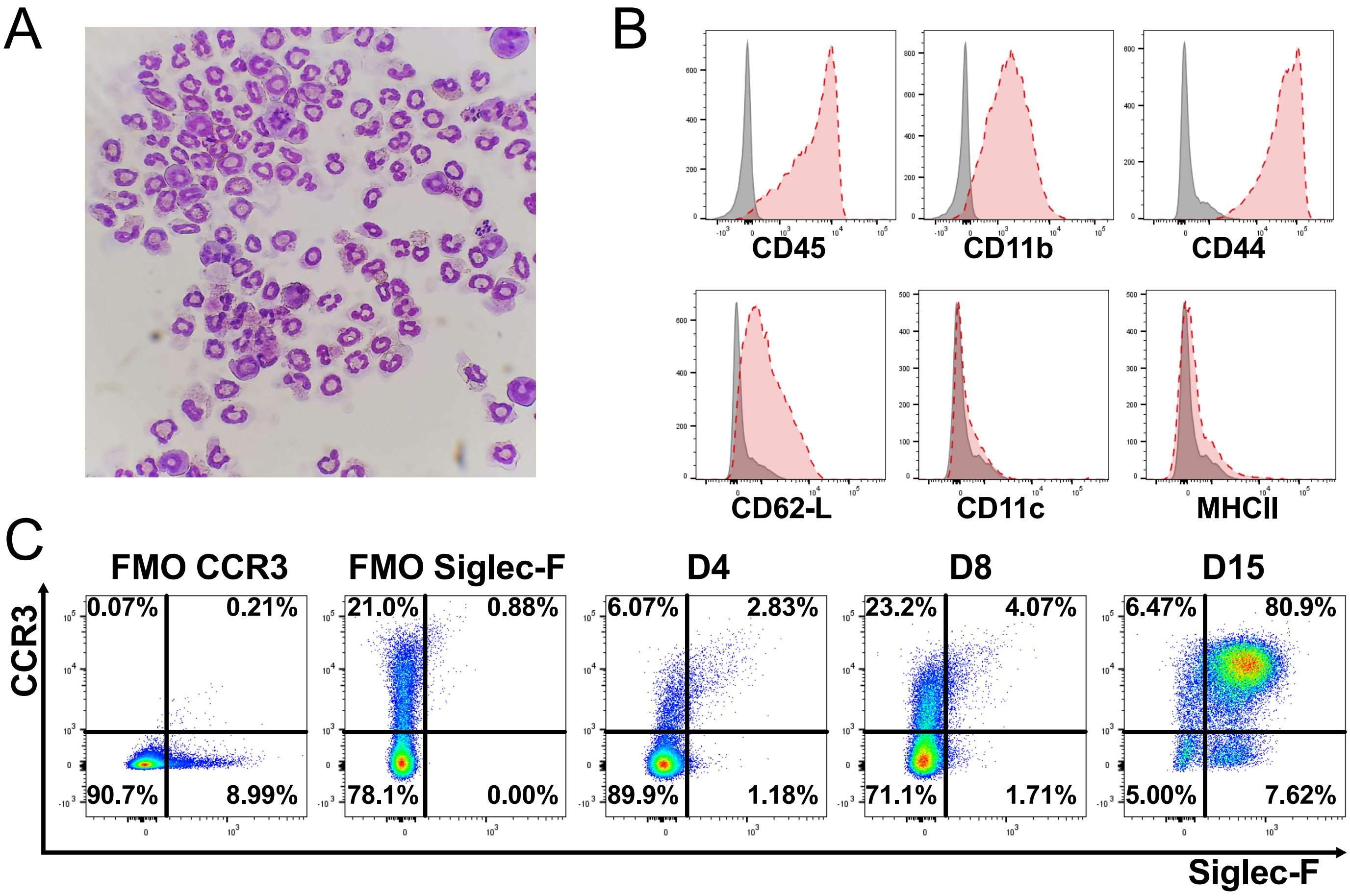


**Figure S1.** Flow cytometry analysis of mouse bone marrow-derived eosinophils (mEOS). (**A**) Morphology of mEOS at day 15 of differentiation determined by DiffQuik staining. (**B**) Histogram of CD45, CD11b, CD44, CD62-L, CD11c and MHCII expression (red) compared to their fluorescence minus one (FMO) control (grey) on mEOS at the end of the differentiation process (day 15). (**C**) Gating strategy based on FMO control and representative flow cytometry profile of mEOS ($CCR3^+$, Siglec-$F^+$) after 4, 8 and 15 days of differentiation.
